# Research on biliary atresia and epigenetic factors from the perspective of transcriptomics: identification of key genes and experimental validation

**DOI:** 10.3389/fped.2025.1624671

**Published:** 2025-07-17

**Authors:** Zhao Na, Hang Yang, Li Chen, Han Xiao, Bo Hai, Chuanxin Li, Xiaohui Xie, Qiang Bai

**Affiliations:** Department of General Surgery, Kunming Children’s Hospital, Children’s Hospital Affiliated to Kunming Medical University, Kunming, China

**Keywords:** biliary atresia, epigenetic factors, bioinformatics, GEO, molecular docking

## Abstract

**Background:**

Biliary atresia (BA) is a severe pediatric liver disease. However, the role of epigenetic factors in its pathogenesis remains poorly understood. This study aimed to identify key genes associated with BA and epigenetic factors, as well as to explore potential therapeutic drugs, thereby offering new insights into the treatment of this condition.

**Methods:**

Transcriptomic datasets (training set GSE122340 and validation set GSE46960) were analyzed. The training set was used to identify differentially expressed genes (DEGs) between BA and normal samples. Candidate genes were selected by intersecting the DEGs with epigenetic factor-related genes. A protein-protein interaction (PPI) network was constructed, and key genes displaying consistent expression patterns across both datasets were identified. Localization, correlation, and Gene Set Enrichment Analysis (GSEA) of these key genes were performed. A molecular regulatory network was constructed, and drug predictions, along with molecular docking simulations, were conducted for the key genes. Experimental validation of the bioinformatics findings was carried out.

**Results:**

A total of 3,462 DEGs were identified, from which 62 candidate genes were selected. Five key genes (AURKA, BUB1, CDK1, RAD51, TOP2A) were highlighted, all of which exhibited strong positive correlations and were linked to essential pathways, including the cell cycle. Thirteen potential drugs were identified, with three pairs showing strong binding affinities. RT-qPCR validation confirmed that, except for CDK1, AURKA, BUB1, RAD51, and TOP2A exhibited consistent trends with the bioinformatics analysis, and were significantly upregulated in the BA group.

**Conclusion:**

This study successfully identified key genes (AURKA, BUB1, CDK1, RAD51, TOP2A) and potential therapeutic drugs for BA, providing critical insights into its pathogenesis and offering potential avenues for novel treatment strategies.

## 1 Introduction

Biliary atresia (BA) is a severe neonatal cholangiopathy characterized by progressive inflammation and fibrosis of both intrahepatic and extrahepatic bile ducts (1). If left untreated, it can lead to bile duct obstruction, liver cirrhosis, and ultimately liver failure (2). Early diagnosis and prompt surgical intervention, particularly the Kasai portoenterostomy, are critical for improving outcomes in infants with BA (3, 4). Despite surgical treatment, BA remains the leading cause of end-stage liver disease and the primary indication for pediatric liver transplantation (1, 3, 5). While substantial research has been conducted, the etiology of BA remains poorly understood, with genetic variants, environmental factors, toxins, viral infections, and immune dysfunction all thought to contribute to its pathogenesis (6–8). Thus, further investigation into the disease mechanisms and potential therapeutic targets for BA is crucial to improving patient outcomes.

Epigenetics refers to heritable changes in gene function that do not involve alterations in the DNA sequence, thereby influencing phenotype (9). Epigenetic mechanisms, including DNA methylation, histone modifications, and chromatin remodeling, play critical roles in regulating gene expression and are implicated in the development of various liver diseases (10–12). Epigenetic factors serve as key regulators of gene expression, bridging genetic and environmental influences (13). Although several studies have examined genetic alterations in BA, the findings remain inconclusive, highlighting the need to explore non-coding and epigenetic factors that may contribute to disease onset (6). Notably, the interaction between genetic and epigenetic predispositions, combined with environmental exposures during pregnancy, may act as triggers for BA (8, 10). Approximately 20% of BA cases are associated with embryonic development, with epigenetic factors playing a pivotal role in disease pathogenesis (14). Epigenetic mechanisms may lead to abnormal expression of proliferation-related genes in BA; however, there is currently no research exploring the specific molecular mechanisms involved. Therefore, further identification of key genes associated with these epigenetic mechanisms is crucial for understanding the specific mechanisms linking BA.

Although research on BA has advanced, the specific epigenetic modifications and their functional implications remain poorly understood. To address this, the present study leverages transcriptomic data from public BA databases to identify key genes associated with epigenetic factors through protein-protein interaction (PPI) analysis. Various bioinformatics techniques will be employed to identify biological pathways related to these key genes. Additionally, molecular regulatory networks will be constructed, and potential therapeutic drugs will be predicted, providing theoretical support for BA treatment. To validate the bioinformatics findings, RT-qPCR experiments will be conducted to assess the mRNA expression levels of key genes, confirming their alignment with computational analyses. These results will offer a deeper understanding of the molecular mechanisms linking BA with epigenetic factors and may contribute to the development of new therapeutic approaches.

## 2 Methods

### 2.1 Data collection

Two datasets, GSE122340 and GSE46960, were retrieved from the GEO database (https://www.ncbi.nlm.nih.gov/geo/). GSE122340 served as the training set, comprising 171 liver tissue samples from patients with BA and 7 liver tissue samples from normal children (NM) (15, 16), with sequencing performed on the GPL16791 platform. GSE46960 was used as the validation set, containing 64 BA liver tissue samples and 7 NM liver tissue samples. Fourteen infants with intrahepatic cholestasis due to other causes and 10 normal adult liver tissue samples were excluded (17). The sequencing platform for GSE46960 was GPL6244. A total of 720 epigenetic factor-related genes (EFGRs) were obtained from the Epifactors database (http://epifactors.autosome.ru/) (18) (Supplementary Table S1).

### 2.2 Analysis of differentially expressed genes (DEGs)

The gene expression matrix from the training set GSE122340 was analyzed using the “DESeq2” (v 1.40.2) (19) to identify DEGs between the BA and NM groups, setting the thresholds as adj. *P*-value < 0.05 and |log_2_FC| > 1, and corrected the *p*-values using the Benjamini-Hochberg (BH) method. The “ggplot2” (v 3.5.1) (20) package was used to generate the volcano plot for visualizing DEGs, while the “ComplexHeatmap” (v 2.16.0) (21) package was utilized to create a heatmap of the top 10 most significantly up- and down-regulated DEGs. Next, the intersection of the DEGs and EFGRs was determined using “ggvenn” (v 0.1.9) (22) to identify candidate genes.

### 2.3 Enrichment analysis

Gene Ontology (GO) and Kyoto Encyclopedia of Genes and Genomes (KEGG) pathway analyses of the candidate genes were performed using “clusterProfiler” (v 4.15.0) (19), with a significance threshold of *P* < 0.05, to explore the biological functions and pathways associated with these genes.

### 2.4 PPI network construction

To examine the interaction relationships between proteins encoded by the candidate genes, the STRING database (http://www/string-db.org/) was used to construct a PPI network, with species set to “Homo sapiens” and an interaction score threshold of ≥0.4. Discrete nodes were excluded from the analysis. Cytoscape software (v 3.10.3) (23) was then employed to visualize the PPI network. Highly connected regions within the network are likely to play a critical role in biological regulation. Gene screening from the PPI network was further refined by evaluating the importance of each node using three different algorithms (MCC, MNC, Degree) via the Cytohubba plugin in Cytoscape. The top 9 genes with the highest scores in each algorithm were selected. The intersection of genes from the three algorithms was identified using “ggvenn” (v 0.1.9) (22), and these genes were classified as core genes. Finally, the expression levels of the core genes in the training set GSE122340 and validation set GSE46960 were analyzed using the Wilcoxon rank-sum test. Genes were considered key when their expression patterns in the training set were consistent with those in the validation set, and when significant differences (*P* < 0.05) between the BA and NM groups were observed.

### 2.5 Localization analysis

Gene localization plays a pivotal role in understanding the structure, function, and interactions of genes. To determine the chromosomal locations of the key genes, their distribution across chromosomes was visualized using the “RCircos” (v 1.2.2) (24) package. To explore the functions of the key genes, their DNA sequences were initially retrieved from the Gene database (https://www.ncbi.nlm.nih.gov/gene/). Subsequently, the subcellular localization of the key genes was predicted using the mRNALocater database (http://bio-bigdata.cn/mRNALocater/). The tissue/organ-specific expression patterns of these genes were analyzed using the BioGPS database (http://biogps.org/), with the following screening criteria: (1) Transcripts of key genes linked to a specific organ system should have expression levels greater than ten times the median value; (2) The expression level of key genes in the second-most abundant tissue should be less than one-third of the highest expression value. Genes meeting these criteria were considered to exhibit tissue/organ specificity.

### 2.6 Exploration of correlation analysis and network construction

To assess the functional similarities among the key genes in BA, a Friends analysis was conducted. This method compares the similarities between genes or gene sets based on functional information. The “GOSemSim” (v 2.33.0) (25) package was used to calculate functional similarity scores, and the results were visualized. To further explore the correlations between key genes in BA, the Spearman correlation coefficient was calculated across all samples in the training set GSE122340 using the “Hmisc” (v 5.1-3) (26) package (|cor| > 0.3, *P* < 0.05). The correlation heatmap was drawn using the “corrplot” (v 0.95) (27) package for visualization. Additionally, the GeneMANIA database (https://genemania.org/) was employed to predict genes related to the functions of the key genes and the biological processes they are involved in. The interaction network between the key genes and the predicted genes was also visualized to uncover additional genes and functions associated with the key genes.

### 2.7 Gene set enrichment analysis (GSEA)

To investigate the biological pathways associated with the key genes in BA, the KEGG gene set “c2.cp.kegg.v7.4.symbols.gm” was retrieved from the MSigDB as a reference gene set. The Spearman correlation coefficients between the key genes and other genes in the GSE122340 training set samples were calculated using the “psych” (v 2.1.6) (28) package, and the genes were ranked in descending order based on these coefficients. GSEA was performed (*P* < 0.05) using the “clusterProfiler” (v 4.15.0) (19) package, with the top 5 most significant pathways being presented based on the *P*-values.

### 2.8 Construction of molecular regulatory network

To explore the regulatory effects on key genes, transcription factors (TFs) interacting with the key genes were predicted using CHIPBase (https://rnasysu.com/chipbase3/), and the TF-gene interactions were sorted based on the number of supporting samples. Additionally, miRNAs interacting with the key genes were predicted using the starBase database (http://starbase.sysu.edu.cn). Finally, the regulatory networks of the top 10 pairs of “TF-key gene” and “miRNA-key gene” interactions for each key gene were visualized using Cytoscape software (v 3.10.3) (23).

### 2.9 Drug prediction and molecular docking

To explore potential drugs for the treatment of BA, the DGIdb database (http://dgidb.org/) was used for drug retrieval. This process identified drugs or molecular compounds potentially associated with the key genes, ultimately pinpointing drugs that might target these genes. For each key gene, the top 10 “drug-key gene” interactions were visualized using Cytoscape software (v 3.10.3) (23). The drugs with the highest Interaction Scores in the DGIdb database (http://dgidb.org/) for each key gene were designated as potential therapeutic agents.

To further elucidate the interaction mechanism between the potential drugs and key genes, the 3D structures of proteins corresponding to the key genes were obtained from the RCSB Protein Data Bank, while the 3D molecular structures of the active ingredients of the potential drugs were retrieved from the PubChem database (https://pubchem.ncbi.nlm.nih.gov/). Molecular docking was performed using the CB-DOCK2 website, with binding ability indicated by binding energy. A binding energy of less than or equal to −5.0 kcal·mol^–1^ suggested an extremely strong binding affinity between the drug and key gene.

### 2.10 Experimental validation

To validate the differences in key gene expression levels, RT-qPCR experiments were conducted. The liver tissue samples used in the experiments were collected from Kunming Children's Hospital, including 5 patients with BA and 5 healthy controls. The study was approved by the Ethics Committee of Kunming Children's Hospital (2025-05-052-K01) and strictly followed the ethical guidelines of the Declaration of Helsinki. Informed consent was obtained from all participants. Total mRNA was extracted using the TRIzol (Vazyme, Nanjing, China) method, and the concentration was measured to ensure an A260/280 ratio between 1.8 and 2.0. A total of 2 µg RNA was reverse transcribed according to the instructions of the HP All-in-one qRT Master Mix II RT203 Ver.1 kit (YunGen Biotechnology, Kunming, China). SYBR Green-based RT-qPCR was subsequently performed using the Hifair® Ⅲ 1st Strand cDNA Synthesis SuperMix for qPCR kit. The relevant primer sequences are shown in Table 1, and GAPDH was used as the internal control gene for data normalization. The 2^–△△CT^ method was used to calculate gene expression levels of the key genes (29).

**Table 1:** Primer sequences for key genes.

Primer	Sequence
AURKA F	TTCCTCCGTCCCTGAGTGT
AURKA R	GGTCCATGATGCCTCTAGC
BUB1 F	TGCAGAGCTACAAGGGCAAT
BUB1 R	CCCAGGCAATGTACAGAGGG
CDK1 F	CGTAGCTGGGCTCTGATTGG
CDK1 R	CAAACTCACCGCGCTAAAGG
RAD51 F	CATCGCCCATGCATCAACAA
RAD51 R	TGGCATCTCCCACTCCATCT
TOP2A F	CAAGAATCGCCGCAAAAGGA
TOP2A R	AGCCACAGCTGAGTCAAAGT
H-GAPDH F	ATGGGCAGCCGTTAGGAAAG
H-GAPDH R	AGGAAAAGCATCACCCGGAG

### 2.11 Statistical analysis

Bioinformatics analyses were performed using the R programming language (v 4.3.3). Gene expression bar charts were created with GraphPad Prism 10, and the t-test was used to compute *P*-values. A *P*-value less than 0.05 was considered statistically significant (****: *P* < 0.0001; ***: *P* < 0.001; **: *P* < 0.01; *: *P* < 0.05; ns: *P* > 0.05).

## 3 Results

### 3.1 Screening and enrichment analysis of candidate genes

**Figure 1:**
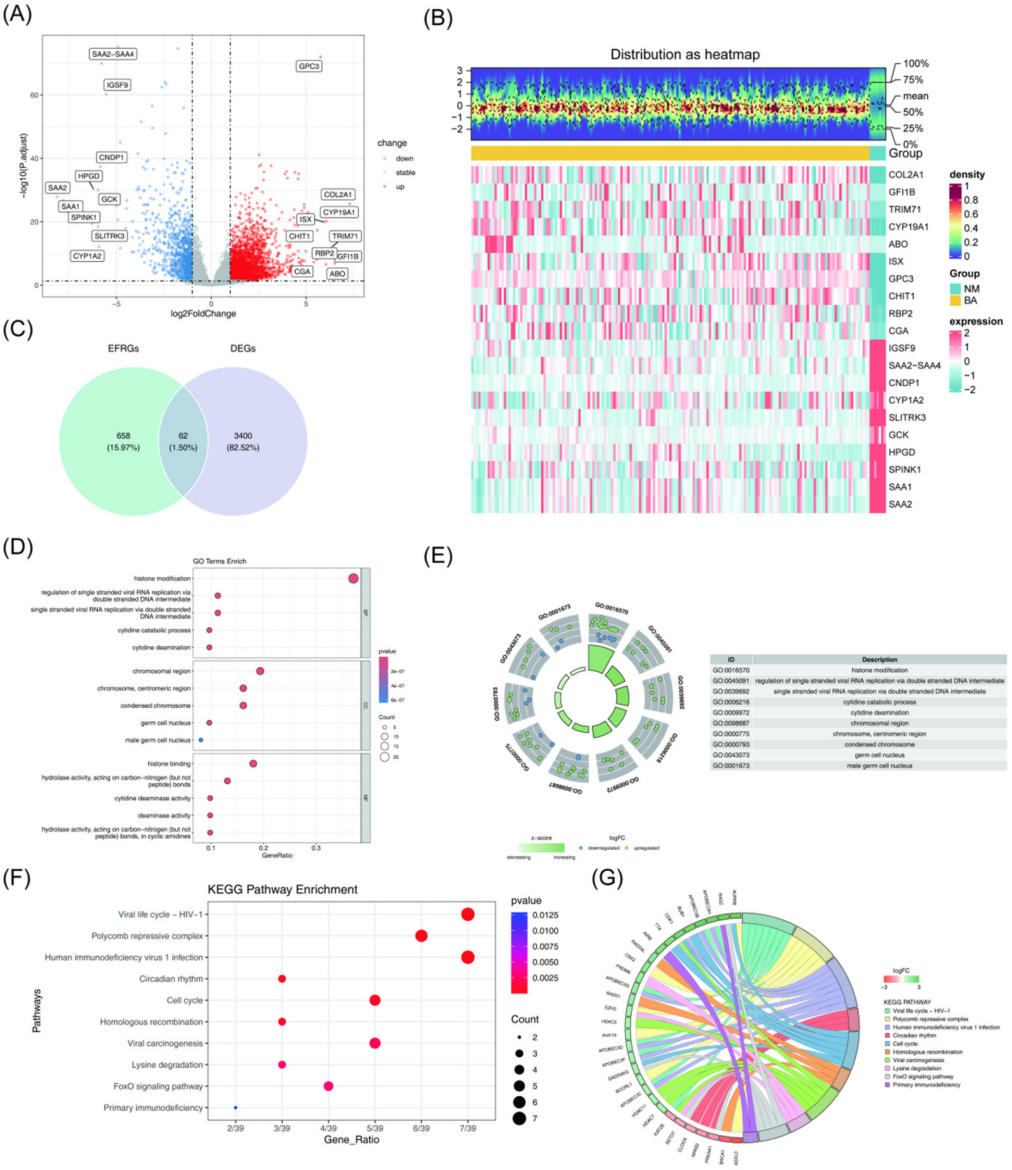
Screening and enrichment analysis of candidate genes. **(A)** Volcano plot displaying the DEGs between the BA group and the NM group. Red plots represent upregulated genes, while blue plots indicate downregulated genes. **(B)** Heatmap showing the DEGs between the BA group and the NM group, where red represents high expression and green represents low expression. **(C)** Venn diagram illustrating the 62 candidate genes identified. **(D)** GO enrichment analysis of candidate genes. In the bubble chart, bubble size represents the number of genes, and color reflects the *P*-value. **(E)** Top 10 biological functions identified by GO. **(F,G)** KEGG pathway analysis of candidate genes. Top 10 pathways enriched by KEGG. The top 10 pathways enriched by KEGG. DEGs, Differentially expressed genes; BA, biliary atresia; NM, normal; GO, gene ontology; KEGG; kyoto encyclopedia of genes and genomes.

The DEGs between the BA and NM groups in the training set GSE122340 were identified using the criteria of adj. *P*-value <0.05 and |log_2_FC| > 1. A total of 3,462 DEGs were detected, with 2,464 genes upregulated and 998 genes downregulated in the BA group compared to the NM group (Figures 1A,B, Supplementary Table S2). To identify genes related to epigenetic factors in BA, the intersection of the 3,462 DEGs and 720 epigenetic factor-related genes (EFRGs) was analyzed, resulting in 62 candidate genes (Figure 1C).

Next, GO and KEGG analyses were performed on the 62 candidate genes to gain insight into their biological functions (*P* < 0.05). GO analysis revealed 349 biological functions. Of these, 269 terms were in the biological process category, primarily enriched in histone modification, cytidine catabolic process, and cytidine deamination. Thirty-three terms were in the cellular component category, with enrichment in chromosomal regions such as the centromeric region, condensed chromosome, germ cell nucleus, and male germ cell nucleus. The remaining 47 terms fell under molecular functions, with significant enrichment in cytidine deaminase activity, histone binding, deaminase activity, and hydrolase activity acting on carbon-nitrogen bonds in cyclic amidines (Figures 1D,E, Supplementary Table S3).

The KEGG pathway analysis identified the top 10 enriched pathways, including the cell cycle, homologous recombination, lysine degradation, FoxO signaling pathway, and primary immunodeficiency pathways (Figures 1F,G, Supplementary Table S4).

Epigenetics involves the regulation of gene expression through mechanisms like DNA methylation and histone modification, which do not alter the underlying DNA sequence. In the GO enrichment analysis, terms related to histone modification and histone binding directly linked to epigenetic regulation were identified. Histone modification, as a key form of epigenetic regulation, can alter chromatin structure and function, ultimately influencing gene expression. Additionally, the viral life cycle (HIV-1) pathway, associated with viral RNA replication, was found to be enriched. Given that viral infections might trigger immune responses leading to biliary epithelial cell damage and subsequently BA, these findings suggest that viral factors may contribute to the onset and progression of BA (30).

### 3.2 Screening of key genes

**Figure 2:**
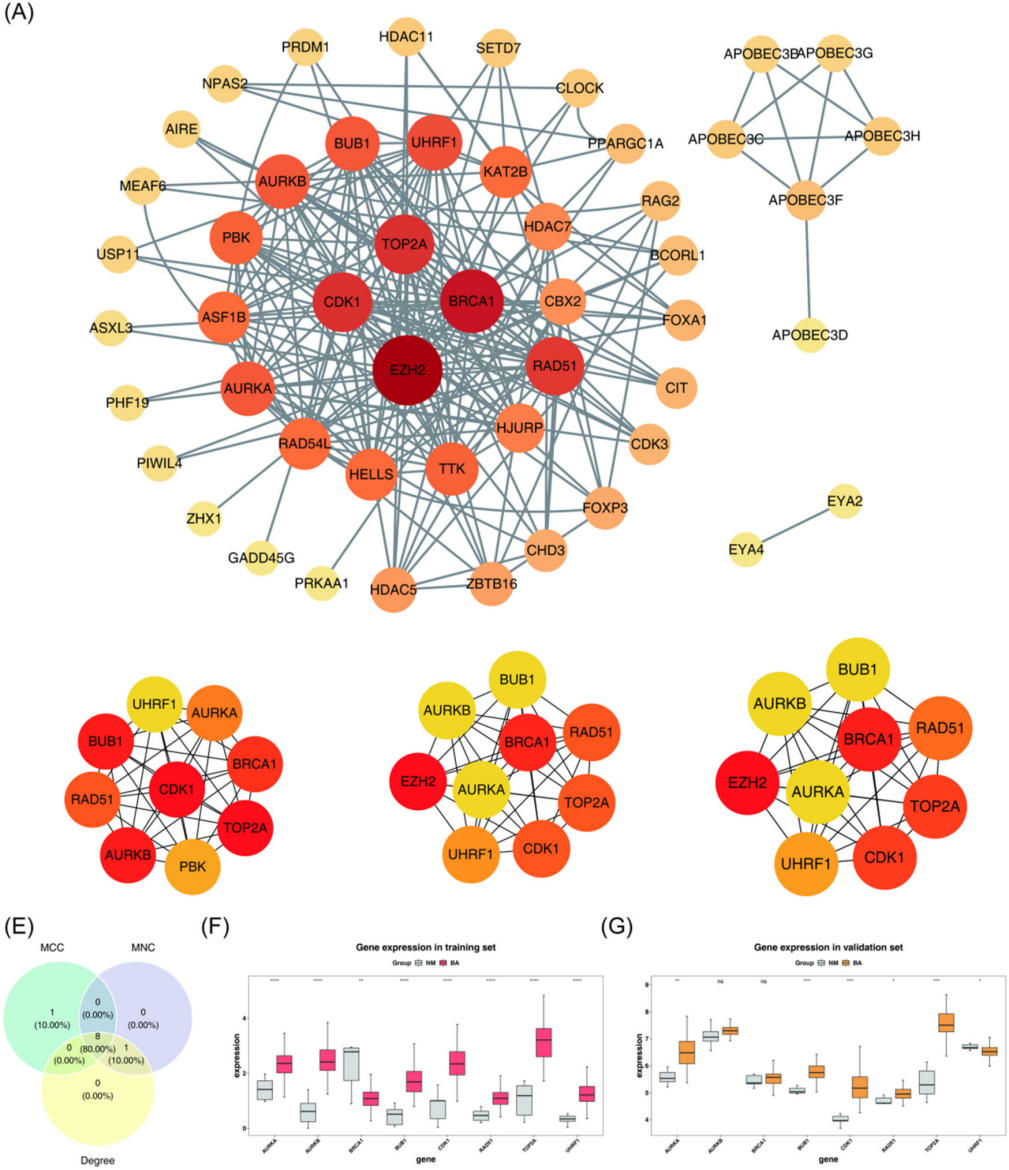
Screening of key genes. **(A)** PPI network of candidate genes. Each label represents a protein, and each line indicates an interaction between proteins. **(B–D)** Top 9 genes with the highest scores identified by three different algorithms: MCC, MNC, and Degree. **(E)** Identification of 8 core genes. **(F,G)** Key genes are significantly upregulated in both the BA group of the training set and the validation set. BA, biliary atresia; NM, normal; PPI, protein-protein interaction; MCC, maximal clique centrality; MNC, maximum neighborhood component. ns, not significant, *P < 0.05, **P < 0.01, ***P < 0.001.

To identify the key genes, a PPI network was constructed to visualize the interactions among proteins encoded by the candidate genes. Isolated genes without interactions were removed, while genes with interactions were retained (Figure 2A). The importance of each gene was assessed using three different algorithms: MCC, MNC, and Degree. The top 9 genes with the highest scores in each algorithm were selected (Figures 2B–D). By intersecting the 9 genes identified by each algorithm, 8 core genes were obtained: TOP2A, CDK1, AURKB, BUB1, BRCA1, RAD51, AURKA, and UHRF1 (Figure 2E).

The expression levels of these 8 core genes were further analyzed. As presented in Figures 2F,G, AURKA, BUB1, CDK1, RAD51, and TOP2A were significantly upregulated in the BA group in both the training set (GSE122340) and the validation set (GSE46960) (*P* < 0.05). These 5 genes were designated as key genes for further analysis.

### 3.3 Analysis of key genes localization and tissue specificity

**Figure 3:**
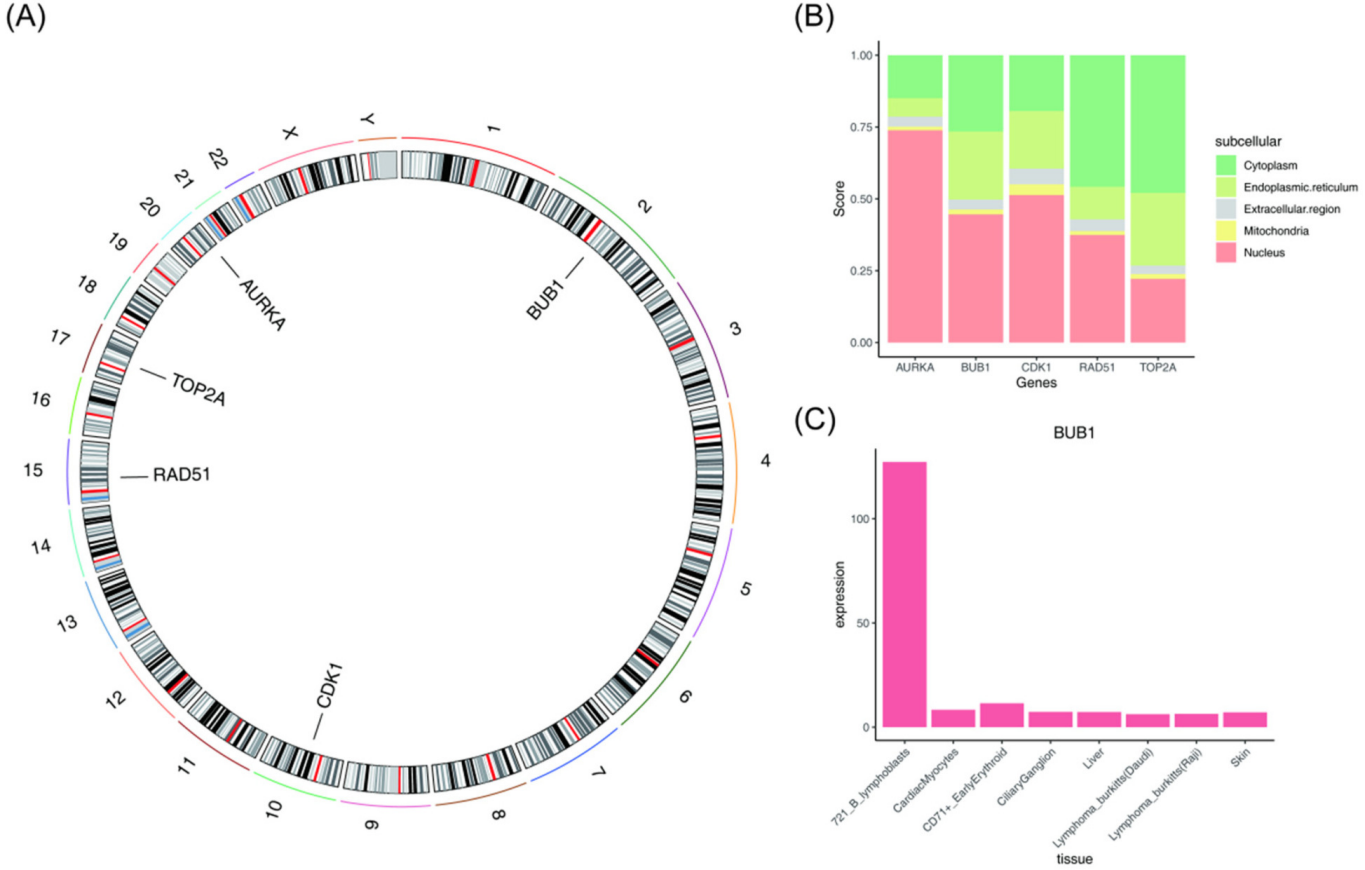
Localization and tissue specificity of key genes (AURKA, BUB1, CDK1, RAD51, and TOP2A). **(A)** Chromosomal localization of the key genes. **(B)** Subcellular localization of the key genes. **(C)** BUB1 exhibits tissue specificity in human lymphocytes (721 B lymphoblasts).

To investigate the localization and tissue specificity of these key genes, chromosome localization, subcellular localization, and tissue specificity were examined. As depicted in Figure 3A, the AURKA gene was located on chromosome 20, BUB1 on chromosome 2, CDK1 on chromosome 10, RAD51 on chromosome 15, and TOP2A on chromosome 17. As depicted in Figure 3B, AURKA, BUB1, and CDK1 were predominantly localized in the nucleus, while RAD51 and TOP2A were primarily found in the cytoplasm. As presented in Figure 3C, BUB1 exhibited tissue specificity in human lymphocytes (721 B lymphoblasts), while the other four key genes did not show clear tissue/organ specificity (Supplementary Figure S1).

### 3.4 Correlation analysis among key genes

**Figure 4:**
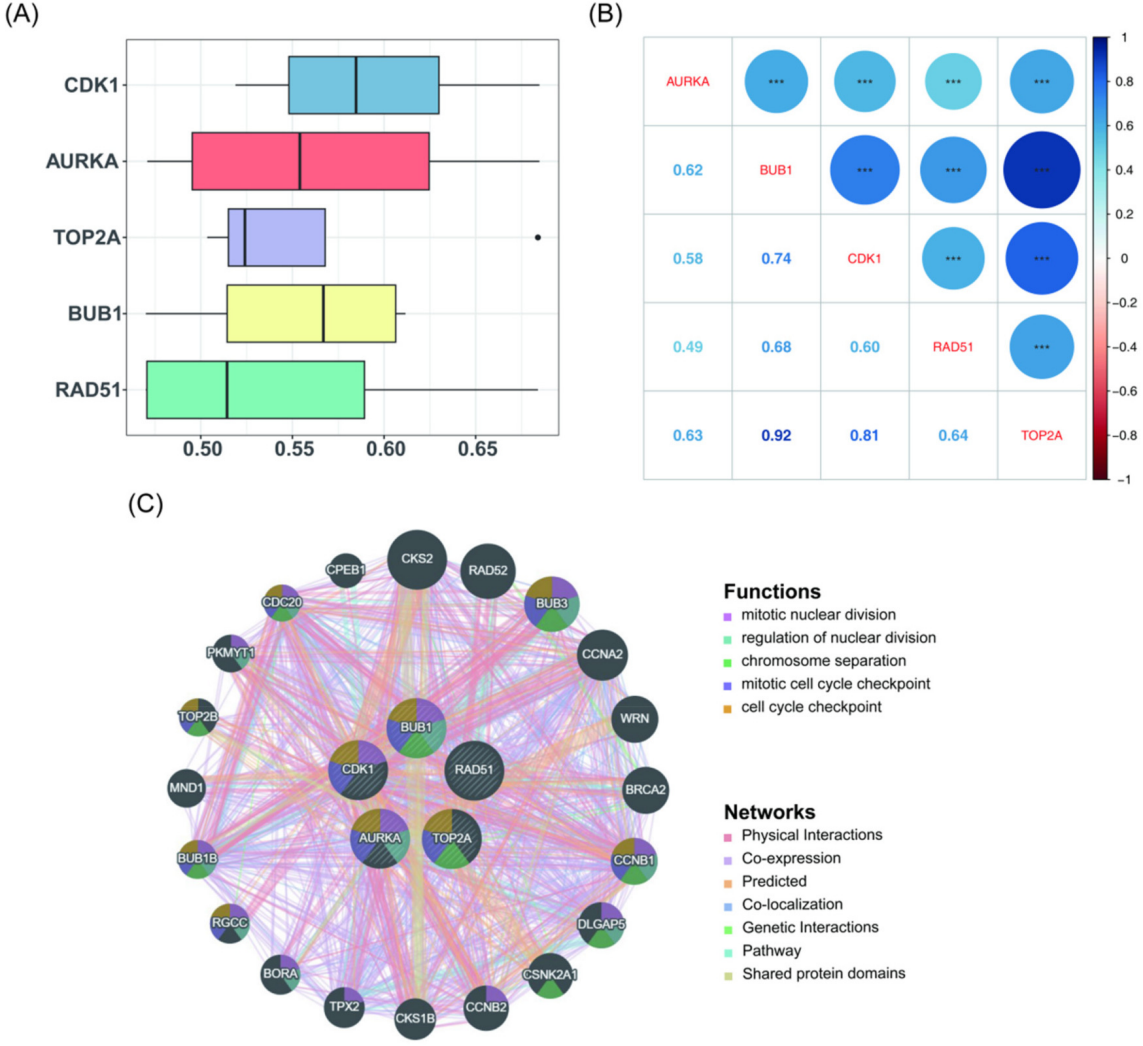
Correlation analysis among key genes (AURKA, BUB1, CDK1, RAD51, and TOP2A). **(A)** Friends analysis indicating that CDK1 exhibits the strongest correlation with the other key genes at the functional level. **(B)** Correlation analysis revealing that BUB1 and TOP2A have the highest correlation, with a coefficient of 0.92. **(C)** Interaction network of key genes and their predicted associated genes. The inner circle consists of the five key genes, while the outer circle contains 20 other genes related to the functions of the key genes.

To explore the relationships among the key genes, a Friends analysis was conducted, which showed that CDK1 exhibited the strongest correlation with the other key genes at the gene function level (Figure 4A). Subsequently, the correlations among the key genes were analyzed using Spearman's correlation (|cor| > 0.3, *P* < 0.05), revealing highly significant positive correlations (*P* < 0.001) among the five key genes. The strongest correlation was observed between BUB1 and TOP2A, with a correlation coefficient of 0.92 (Figure 4B). Further exploration of genes associated with the key genes and their functions was conducted using the GeneMANIA database. Figure 4C illustrates a network in which the five key genes form the inner circle, while 20 other genes related to their functions are positioned in the outer circle. These genes interact with one another through seven distinct interaction types and are involved in five key biological processes: mitotic nuclear division, regulation of nuclear division, chromosome separation, mitotic cell cycle checkpoint, and cell cycle checkpoint. Epigenetic factors such as DNA methylation and histone modification may regulate the expression of these genes, influencing the cell cycle and division processes, which could impair normal bile duct development and increase the risk of BA development.

### 3.5 GSEA

**Figure 5:**
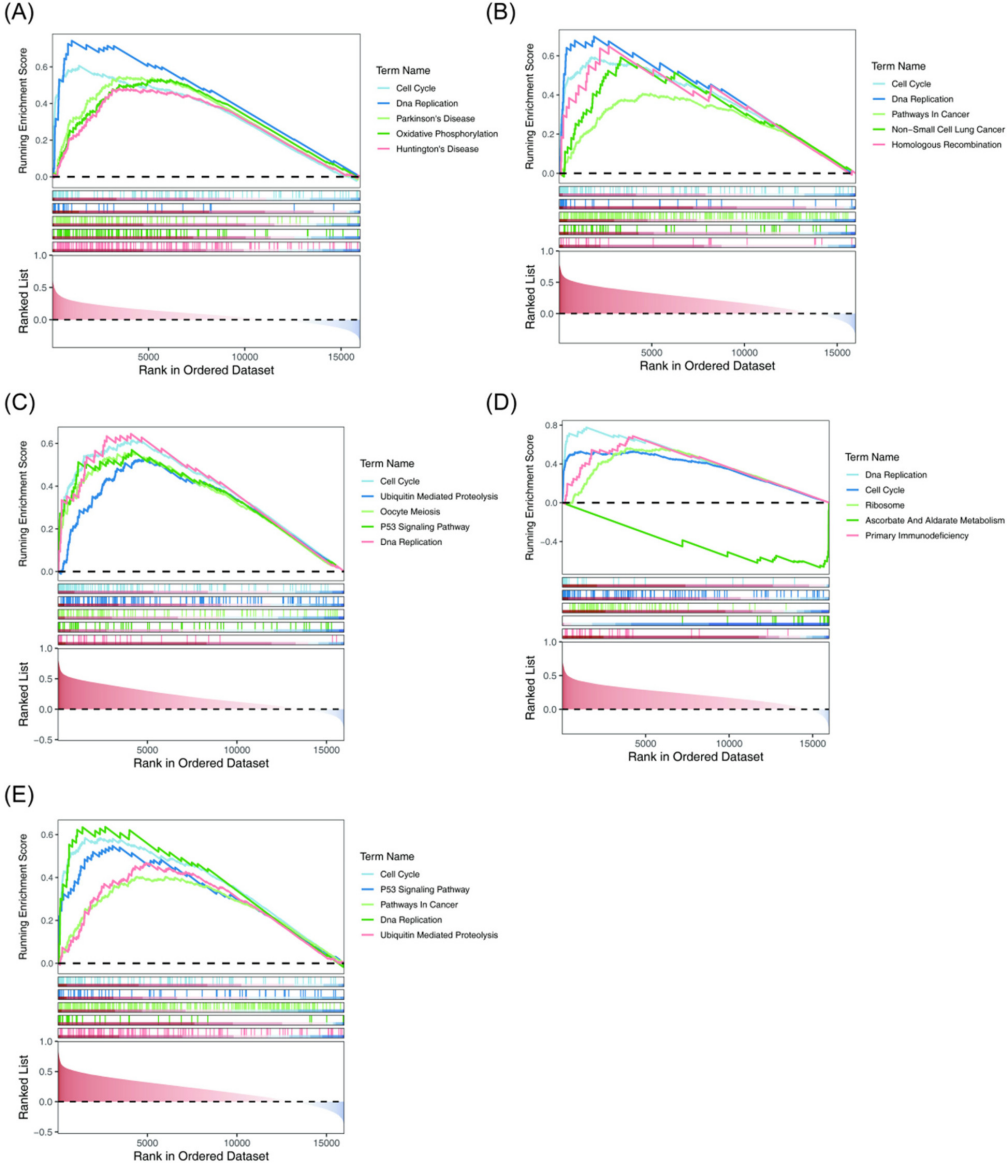
GSEA of the key genes. Both the Cell Cycle and DNA replication pathways were significantly positively correlated with the five key genes. **(A)** AURKA. **(B)** BUB1. **(C)** CDK1. The P53 Signaling Pathway showed a significant positive correlation with CDK1. **(D)** RAD51. Primary Immunodeficiency exhibited a significant positive correlation with RAD51. **(E)** TOP2A. The P53 Signaling Pathway demonstrated a significant positive correlation with TOP2A. GSEA, gene set enrichment analysis.

To investigate the biological pathways involving the key genes in BA, GSEA was conducted, with results presented in Figure 5. Both the Cell Cycle and DNA Replication pathways exhibited significant positive correlations with the five key genes, suggesting that these pathways may have been aberrantly activated during the progression of BA. This activation likely resulted from the synergistic effects of the key genes, disrupting the normal development of biliary epithelial cells. Furthermore, epigenetic modifications of these key genes could have further exacerbated BA development by regulating the cell cycle and DNA replication processes. The P53 Signaling Pathway showed a significant positive correlation with CDK1 and TOP2A, while Primary Immunodeficiency was positively correlated with RAD51. Additionally, Ascorbate and Aldarate Metabolism was negatively correlated with RAD51. These findings imply that in BA, epigenetic regulation of these correlations may influence biliary epithelial cell proliferation, immune responses, and metabolic processes, potentially contributing to the disease's onset and progression.

### 3.6 Construction of the molecular regulatory network of key genes

**Figure 6:**
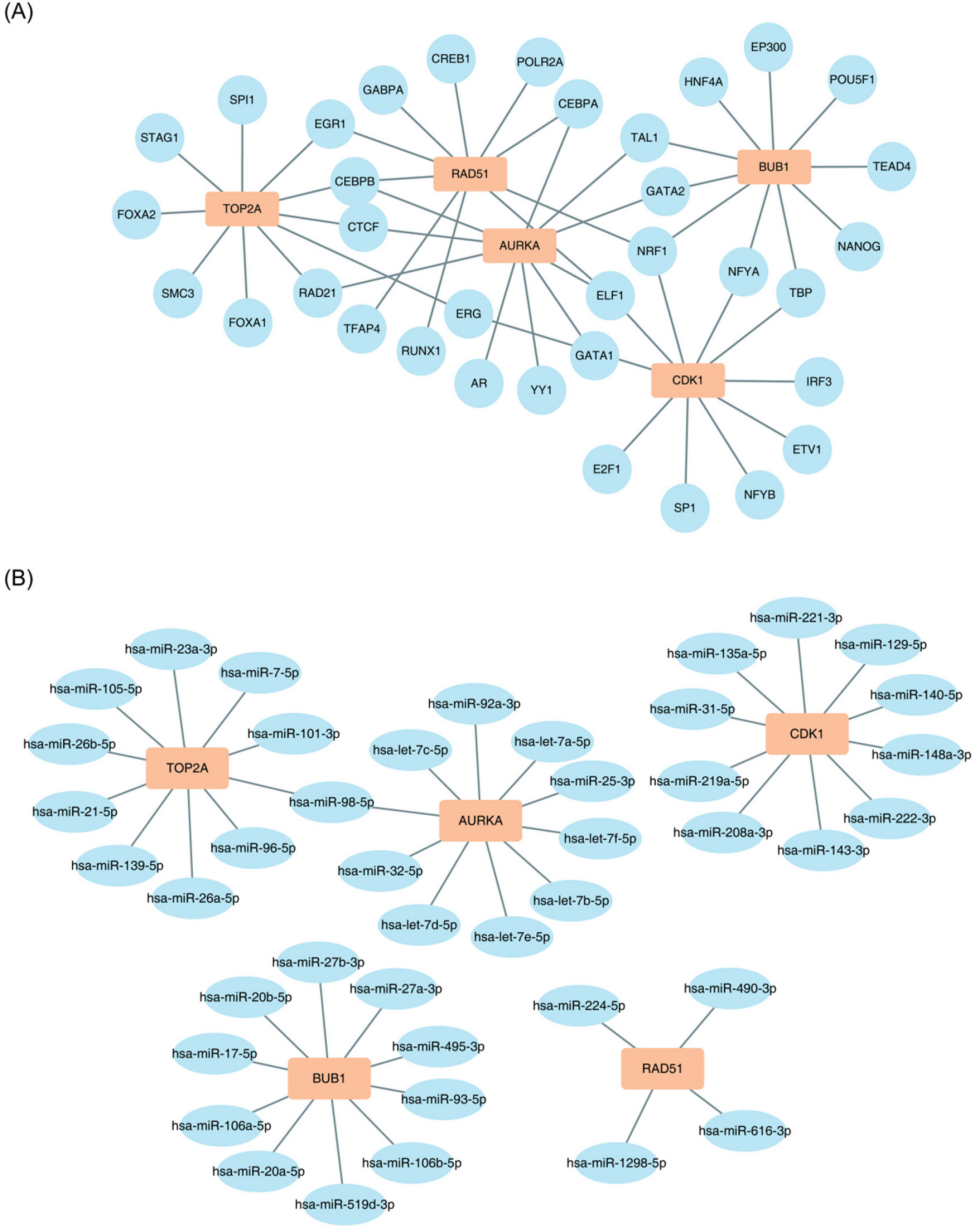
Construction of the molecular regulatory network of key genes. **(A)** TOP2A, RAD51, and AURKA are regulated by CEBPB; RAD51, CDK1, and BUB1 are regulated by NRF1; and AURKA, RAD51, and CDK1 are regulated by ELF1. **(B)** TOP2A and AURKA are regulated by hsa-miR-98-5p.

To examine the regulatory impact of TFs on key genes, predictions were made using the CHIPBase database. As presented in Figure 6A, CEBPB regulates TOP2A, RAD51, and AURKA; NRF1 regulates RAD51, CDK1, and BUB1; and ELF1 regulates AURKA, RAD51, and CDK1.

Similarly, the regulatory influence of miRNAs on key genes was assessed using the starBase database. As presented in Figure 6B, hsa-miR-98-5p may regulate both TOP2A and AURKA.

### 3.7 Drug prediction and molecular docking analysis of key genes

**Figure 7:**
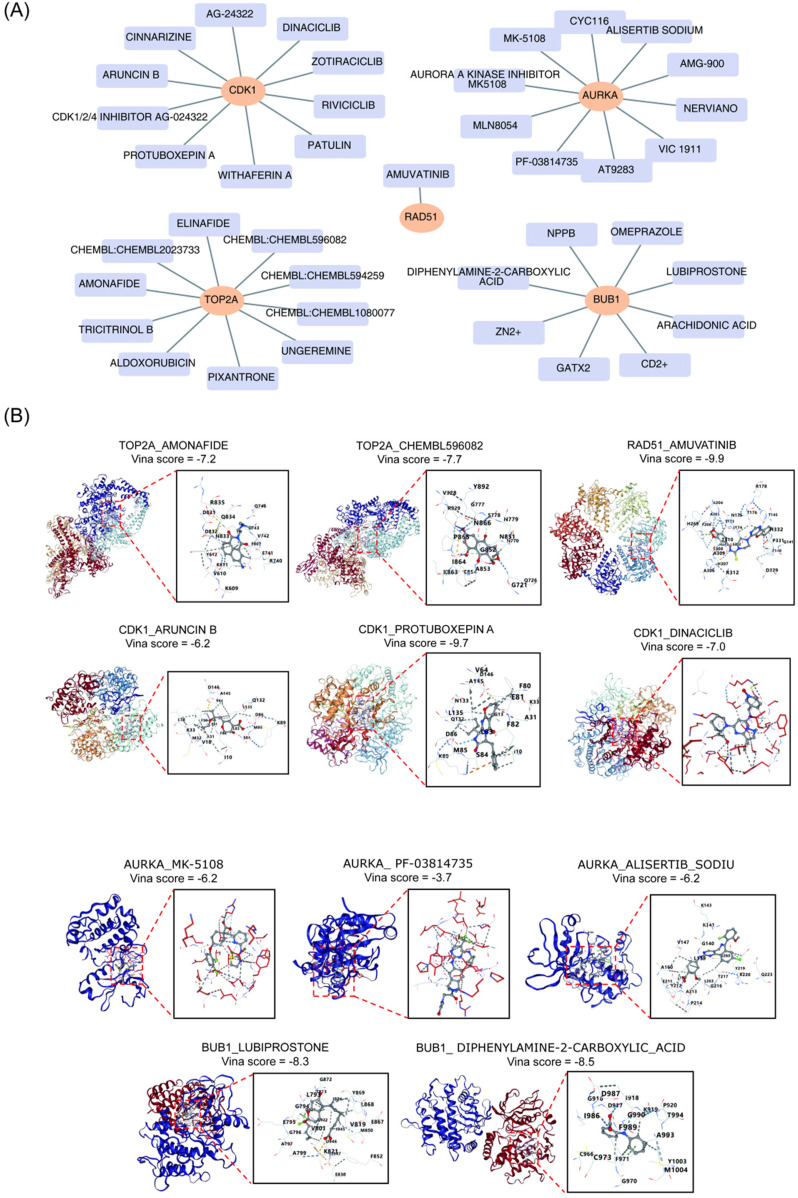
Drug prediction and molecular docking analysis of key genes. **(A)** Top 10 pairs of drug-key gene interactions. **(B)** Molecular docking and score of key genes and potential drugs.

To identify potential drugs for the treatment of BA, drug prediction targeting the five key genes was performed using the DGIdb database. Figure 7A presents the top 10 drug-key gene interaction pairs. The top 3 potential drugs for each key gene, based on the highest Interaction Scores, were selected. For AURKA, the potential drugs were MK-5108 (Interaction Score = 1.80), PF-03814735 (Interaction Score = 1.35), and Alisertib Sodium (Interaction Score = 0.9). For BUB1, the potential drugs were GaTx2 (Interaction Score = 6.53), Lubiprostone (Interaction Score = 3.26), and Diphenylamine-2-carboxylic acid (Interaction Score = 2.17). For CDK1, the potential drugs were Aruncin B (Interaction Score = 2.08), Protuboxepin A (Interaction Score = 1.04), and Dinaciclib (Interaction Score = 0.43). For RAD51, Amuvatinib (Interaction Score = 14.91) was identified as the potential drug. For TOP2A, the potential drugs were Amonafide (Interaction Score = 1.56), ALDOXORUBICIN (Interaction Score = 1.34), and CHEMBL596082 (Interaction Score = 0.89).

Molecular docking simulations were then performed for the 13 potential drugs and their corresponding key genes. Due to the excessive number of atoms and the complexity of their structures, two drugs, ALDOXORUBICIN and GaTx2, could not have their 3D molecular structures generated. As shown in Figure 7B, the strongest binding effects were observed between Amuvatinib and RAD51 (−9.9 kcal·mol^–1^), Protuboxepin A and CDK1 (−9.7 kcal·mol^–1^), and Diphenylamine-2-carboxylic acid and BUB1 (−8.5 kcal·mol^–1^), suggesting that these three drugs may have significant therapeutic potential for BA.

### 3.8 Validation by RT-qPCR experiment

**Figure 8:**
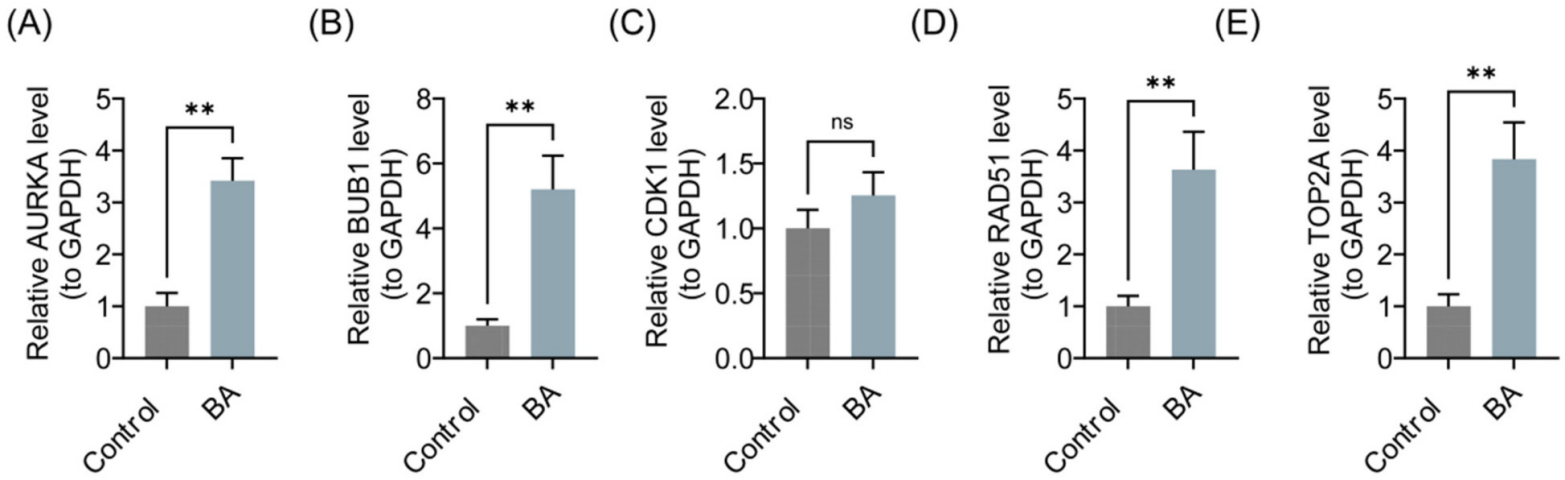
Expression levels of AURKA, BUB1, CDK1, RAD51, and TOP2A in the BA group and the control group. **(A)** AURKA. **(B)** BUB1. **(C)** CDK1. **(D)** RAD51. **(E)** TOP2A. BA, biliary atresia. ns, not significant, *P < 0.05, **P < 0.01, ***P < 0.001.

To validate the consistency of key gene expression levels between clinical tissue samples and bioinformatics results, RT-qPCR experiments were conducted. As presented in Figure 8, compared to the control group, the expression levels of AURKA, BUB1, CDK1, RAD51, and TOP2A were significantly upregulated in the BA group (*P* < 0.01), consistent with the bioinformatics analysis. However, no significant difference was observed in the expression of CDK1 (*P* > 0.05), which might be attributed to sample heterogeneity and small sample size.

## 4 Discussion

BA is not a singular disease with a single cause, but rather a phenotype resulting from multiple pathogenic factors and mechanisms (31). Increasing evidence suggests that patients with BA exhibit a genetic predisposition to heightened biliary injury and pathological repair (1). Recent studies have identified various genetic loci and potential epigenetic factors that may contribute to its pathogenesis, highlighting the complexity of the disease (6). The inconsistent presentation of BA in twins, particularly monozygotic twins, underscores the significant role of epigenetic factors in its development (32). This study identified five key genes—AURKA, BUB1, CDK1, RAD51, and TOP2A—that are linked to epigenetic factors in BA through comprehensive bioinformatics analyses. The functions of these genes were explored using GESA and molecular regulatory networks. Additionally, thirteen potential therapeutic drugs were screened through drug prediction, identifying the strongest binding interactions. Finally, the expression of these key genes was experimentally validated, offering new perspectives for the research and treatment of BA.

AURKA (Aurora Kinase A) is a protein kinase critical for cell mitosis, regulating various aspects of cell division, including centrosome maturation and spindle assembly (33, 34). Dysregulated expression or activity of AURKA is strongly associated with the development of various cancers (35, 36). AURKA is also significantly involved in epigenetic regulation and liver diseases. In cholangiocarcinoma, the TF FOSL1 modulates AURKA expression, influencing cell proliferation and tumor growth (37). Furthermore, AURKA is implicated in cell cycle regulation and epigenetic modifications (38), illustrating the complex regulatory roles of AURKA in liver disease development (39). The upregulation of AURKA can drive the abnormal proliferation of biliary epithelial cells, potentially disrupting bile duct development and function (40). Consequently, AURKA represents a promising target for therapeutic intervention, as modulating its activity could restore normal cell cycle regulation and mitigate the pathological processes underlying BA.

BUB1 (Budding uninhibited by benzimidazoles 1) is integral in monitoring the connection between spindle microtubules and chromosome kinetochores during mitosis (41, 42). Elevated BUB1 levels may serve as a compensatory response to errors in cell division or genomic instability (43). Previous studies have linked high BUB1 expression to aneuploidy (44), a condition that exacerbates the development of various diseases, including human sarcoma (45). Additionally, BUB1 expression in human lymphocytes suggests its involvement in immune responses (46), which may play a significant role in BA, where immune-mediated damage to biliary epithelial cells contributes to disease progression (47). Targeting BUB1 could provide therapeutic benefits by addressing both cell cycle dysregulation and immune-related factors in BA.

CDK1 (Cyclin-dependent kinase 1) is a key regulatory factor in the cell cycle (48), closely related to the transition of cells from the G2 phase to the M phase (49). Dysregulation of CDK1 may lead to uncontrolled cell proliferation (50), which subsequently impairs normal cell differentiation and function. Elevated levels of CDK1 typically indicate abnormal activation of cell cycle regulatory pathways (51). This study found that CDK1 is significantly upregulated in BA liver tissue through transcriptome analysis, suggesting that it may accelerate disease progression by driving cholangiocyte cell cycle dysregulation. Notably, CDK1 is also involved in multiple signaling pathways related to epigenetic regulation, including pathways such as the Wnt/β-catenin signaling cascades. Its activity may be influenced by epigenetic modifications, such as histone acetylation and methylation (52), which further affect gene expression and cell function. However, RT-qPCR results showed no significant differences in CDK1 expression between BA and control samples. This discrepancy may be due to heterogeneity among RT-qPCR samples, including differences in sample source and quality. Additionally, the small sample size may have limited the detection of significant differences. Therefore, future research should explore the molecular interaction mechanisms between CDK1 and epigenetic factors in BA to better understand the epigenetic mechanisms underlying cell cycle dysre.

RAD51, a recombinase essential for homologous recombination, is a core factor in the homologous recombination repair of DNA. It is highly expressed in rapidly proliferating tissues such as the testes, involved in germ cell development, and the colon, responsible for intestinal epithelial renewal, consistent with its role in maintaining genomic stability. RAD51 plays a key role in repairing DNA double-strand breaks and maintaining genomic stability (53, 54). Notably, RAD51 can also influence the activity of DNA methyltransferase DNMT1 by regulating the ubiquitin ligase UHRF1 through two distinct mechanisms, thereby participating in the maintenance of genomic DNA methylation (55). Moreover, in cancer cell lines, RAD51 is associated with the regulation of the autophagy pathway and works together with E-box proteins such as USF1, USF2, and MITF to regulate the expression of autophagy-related genes (56). Autophagy itself is crucial in cellular stress responses and cell death processes (57). In this study, the upregulation of RAD51 in BA suggests that it may indirectly affect the differentiation or function of cholangiocytes by influencing DNA damage repair, modulating the autophagy pathway, and maintaining DNA methylation. Therefore, further research on the specific role of RAD51 in BA will help reveal the molecular mechanisms of this disease and provide a theoretical basis for developing new prevention and treatment strategies.

TOP2A is highly expressed in 721_B lymphoblasts (leukemia cells) and in the testis. It encodes DNA topoisomerase IIα, which is essential for DNA replication and chromosome segregation. Although its overexpression is associated with poor prognosis in various cancers (58–61), the expression of TOP2A is tightly regulated by epigenetic mechanisms, particularly DNA methylation (62). High methylation of its promoter typically suppresses its expression, while low methylation promotes its expression. We found that TOP2A is upregulated in BA, suggesting that there is likely low methylation of the promoter. This finding is consistent with previous observations that low DNA methylation in zebrafish plays a key role in biliary defects (63, 64). Therefore, this epigenetic dysregulation disrupts the tissue-specific expression of TOP2A, leading to its aberrant activation in cholangiocytes and resulting in genomic instability and abnormal cell proliferation in BA cholangiocytes.

GSEA analysis indicates that the pathogenesis of BA is closely linked to the abnormal activation of the cell cycle and DNA replication pathways. Abnormal regulation of the cell cycle pathway strongly correlates with the five key genes, including CDK1, suggesting that these genes work synergistically to promote cell cycle dysregulation. Dysregulation of cell cycle control has been shown to cause excessive proliferation or prevent differentiation in cholangiocytes, leading to disruption of the normal bile duct structure (65). As a key regulator of the cell cycle, CDK1 overexpression accelerates the G2/M phase transition by phosphorylating downstream target proteins, such as retinoblastoma (Rb) protein, which drives uncontrolled cholangiocyte proliferation (66). Moreover, epigenetic modifications, including long non-coding RNAs, may exacerbate cell cycle dysregulation by modulating CDK1 expression (67). This abnormal proliferative state contributes to bile duct fibrosis, which is consistent with the progressive bile duct injury and liver fibrosis seen in patients with BA (65). The significant activation of the DNA replication pathway suggests DNA replication stress and cholangiocyte damage, pointing to potential genomic instability in BA. DNA replication stress can result in replication fork stalling and DNA double-strand breaks (68). This activates the ATR/CHK1 signaling pathway, which may induce apoptosis or cellular senescence (69). The positive correlation between RAD51 and primary immunodeficiency observed in this study may reflect a compensatory enhancement of DNA repair mechanisms in response to sustained replication stress. However, when DNA repair capacity is overwhelmed, accumulated DNA damage may trigger apoptosis through the p53 pathway (70). The negative correlation between the ascorbic acid metabolism pathway and RAD51 suggests that oxidative stress may exacerbate DNA damage. An imbalance in ascorbic acid, a key antioxidant, could reduce cellular defenses against oxidative injury (71). The interaction between these pathways and disease progression indicates that abnormalities in the cell cycle and DNA replication create a vicious cycle. Specifically, an accelerated cell cycle increases the demand for DNA replication, resulting in replication stress (72), while DNA damage can suppress the cell cycle through CDK inhibitors like p21 (73). This imbalance likely explains the simultaneous occurrence of abnormal proliferation and increased apoptosis in cholangiocytes in BA (74). Furthermore, epigenetic modifications, such as irregularities in DNA methyltransferases, may amplify the pathological effects of these pathways by regulating multiple key genes at once (75). In summary, the results from GSEA emphasize the critical role of the cell cycle and DNA replication pathways in the pathogenesis of BA. Disruptions in these pathways may drive dysregulated cholangiocyte proliferation, genomic instability, and metabolic disorders through an epigenetic regulatory network, ultimately leading to the progressive destruction of the bile ducts.

The constructed molecular regulatory network reveals that TFs like CEBPB and miRNAs such as hsa-miR-98-5p regulate key genes. CEBPB is a multifunctional TF involved in regulating inflammation, cell growth, and differentiation (76). CEBPB may serve as a critical link between inflammatory responses and abnormal cell proliferation (76). The presented study identified CEBPB as a regulator of RAD51 and AURKA, suggesting its involvement in the pathological processes of BA by influencing cell proliferation and genomic stability. miR-98-5p inhibits hepatic stellate cell (HSC) activation by directly targeting key factors in the TGF-β signaling pathway, thereby slowing liver fibrosis progression (77). In the present study, hsa-miR-98-5p targeted both TOP2A and AURKA, indicating its potential to protect against BA by inhibiting fibrosis and maintaining genomic stability. However, further experiments are needed to validate the specific regulatory mechanisms of TFs like CEBPB and miRNAs such as hsa-miR-98-5p in the context of BA.

Identifying therapeutic drugs that target key genes associated with BA marks a significant advancement in developing effective treatment strategies. Among the predicted drugs interacting with key genes, Amuvatinib stands out for its strong binding affinity to RAD51, with a docking score of −9.9 kcal/mol. Amuvatinib selectively inhibits receptor tyrosine kinases and has been primarily studied for its efficacy against various cancers, where it impedes tumor growth and induces cancer cell apoptosis (78). Studies suggest that Amuvatinib can reduce tumor cell drug resistance by inhibiting RAD51 protein expression (79, 80). Although the role of RAD51 in BA remains unexplored, it may contribute to cholangiocyte function by regulating DNA repair. Additionally, Protuboxepin A, which targets CDK1, exhibits a docking score of −9.7 kcal/mol. CDK1 is a key regulator of the cell cycle, and its dysfunction is associated with various cancers (81). Protuboxepin A binds directly to α,β-tubulin, stabilizing tubulin polymerization and disrupting microtubule dynamics. This disruption leads to chromosome misalignment and metaphase arrest, ultimately causing apoptosis in cancer cells (81). While the mechanisms of action of Protuboxepin A and CDK1 in BA have not been reported, Protuboxepin A presents a theoretical foundation for potential therapeutic intervention. These findings are crucial not only for identifying individual drug-gene interactions but also for exploring the possibility of combination therapies that target multiple pathways in BA's pathogenesis. However, the aforementioned drugs are currently in the experimental research stage as anticancer agents, and there is still a lack of clinical data supporting their safety, optimal dosage, and potential toxicity in the pediatric population, especially in BA infants. In light of this, future research should prioritize conducting *in vivo* experiments using a BA mouse model induced by rhesus monkey rotavirus. These studies should systematically evaluate the efficacy and safety of the drugs, while optimizing dosing strategies based on the pharmacokinetic characteristics of neonates.

However, this study has several limitations. First, the analysis results depend on data quality, algorithm accuracy, and methodological assumptions. These factors may lead to data noise and difficulties in interpretation due to algorithm complexity; they can also cause issues such as false positives and false negatives. Moreover, the imbalance between the samples of the disease group and the control group may increase the risk of false positives and false negatives. Additionally, although validation was performed through RT-qPCR, the small sample size and differences in experimental methods limited the statistical power to significantly detect the expression pattern of CDK1. To address these issues, future plans include expanding the sample sizes of both the disease group and the control group, as well as optimizing the sample selection strategy to improve the stability and reliability of the research findings. At the same time, we plan to further validate the research findings through experimental techniques such as immunohistochemistry and Western blot. These will be combined with animal models and functional experiments like overexpression and deletion to enhance the credibility of the research conclusions.

## 5 Conclusion

This study identified five key genes (AURKA, BUB1, CDK1, RAD51, TOP2A) related to epigenetic regulation in BA through transcriptomic analysis, whose abnormal expression may promote the progression of BA by interfering with the cell cycle and DNA repair pathways. These genes may serve as potential therapeutic targets for BA, and small molecule inhibitors or antibody drugs targeting them could be developed in the future, which could be further explored for their therapeutic potential. Additionally, this study discovered potential drugs such as Amuvatinib and Protuboxepin A, which may exert therapeutic effects by targeting key genes, providing new hope for the treatment of BA. Future research will focus on investigating the specific regulatory mechanisms of epigenetic modifications (such as DNA hypomethylation) on key genes in animal models, and further experiments will validate the feasibility of these genes as therapeutic targets.

## Data Availability

The datasets presented in this study can be found in online repositories. The names of the repository/repositories and accession number(s) can be found in the article/Supplementary Material.
